# Controlled trial of lovastatin combined with an open-label treatment of a parent-implemented language intervention in youth with fragile X syndrome

**DOI:** 10.1186/s11689-020-09315-4

**Published:** 2020-04-22

**Authors:** Angela John Thurman, Laura A. Potter, Kyoungmi Kim, Flora Tassone, Amy Banasik, Sarah Nelson Potter, Lauren Bullard, Vivian Nguyen, Andrea McDuffie, Randi Hagerman, Leonard Abbeduto

**Affiliations:** 1 MIND Institute, University of California Davis Health, 2825 50th Street, Room 2335, Sacramento, CA 95817 USA; 2 Department of Psychiatry and Behavioral Sciences, University of California Davis Health, Sacramento, USA; 3 Department of Pediatrics, University of California Davis Health, Sacramento, USA; 4 Department of Public Health Sciences, University of California Davis Health, Sacramento, USA; 5 Department of Human Ecology, University of California Davis, Davis, USA; 6 Department of Biochemistry and Molecular Medicine, University of California Davis Health, Sacramento, USA

**Keywords:** Fragile X syndrome, Lovastatin, Distance teleconferencing, Expressive language sampling, Narrative storytelling, Parent-implemented language intervention, PILI

## Abstract

**Background:**

The purpose of this study was to conduct a 20-week controlled trial of lovastatin (10 to 40 mg/day) in youth with fragile X syndrome (FXS) ages 10 to 17 years, combined with an open-label treatment of a parent-implemented language intervention (PILI), delivered via distance video teleconferencing to both treatment groups, lovastatin and placebo.

**Method:**

A randomized, double-blind trial was conducted at one site in the Sacramento, California, metropolitan area. Fourteen participants were assigned to the lovastatin group; two participants terminated early from the study. Sixteen participants were assigned to the placebo group. Lovastatin or placebo was administered orally in a capsule form, starting at 10 mg and increasing weekly or as tolerated by 10 mg increments, up to a maximum dose of 40 mg daily. A PILI was delivered to both groups for 12 weeks, with 4 activities per week, through video teleconferencing by an American Speech-Language Association-certified Speech-Language Pathologist, in collaboration with a Board-Certified Behavior Analyst. Parents were taught to use a set of language facilitation strategies while interacting with their children during a shared storytelling activity. The main outcome measures included absolute change from baseline to final visit in the means for youth total number of story-related utterances, youth number of different word roots, and parent total number of story-related utterances.

**Results:**

Significant increases in all primary outcome measures were observed in both treatment groups. Significant improvements were also observed in parent reports of the severity of spoken language and social impairments in both treatment groups. In all cases, the amount of change observed did not differ across the two treatment groups. Although gains in parental use of the PILI-targeted intervention strategies were observed in both treatment groups, parental use of the PILI strategies was correlated with youth gains in the placebo group and not in the lovastatin group.

**Conclusion:**

Participants in both groups demonstrated significant changes in the primary outcome measures. The magnitude of change observed across the two groups was comparable, providing additional support for the efficacy of the use of PILI in youth with FXS.

**Trial registration:**

US National Institutes of Health (ClinicalTrials.gov), NCT02642653. Registered 12/30/2015.

## Background

Fragile X syndrome (FXS) is the most common inherited cause of intellectual disability and the most common single-gene cause of autism spectrum disorder (ASD) [1, 2]. FXS typically results from a trinucleotide repeat (CGG) expansion mutation, with a consequent transcriptional silencing of the *FMR1* gene and reduction of the encoded protein, fragile X mental retardation protein (FMRP) [3, 4]. FMRP acts as a translational repressor for a number of mRNAs that are important for synaptic functioning and experience-dependent learning [5, 6]. Importantly, the downstream impact of these changes, such as the elevation of basal protein synthesis of an extracellular signal kinase (ERK1/2) signaling pathway, has also been related to the regulation of learning and social behaviors [7–10].

Clinical trials in FXS have been largely unsuccessful despite strong preclinical data suggesting phenotypic improvement even in adult models [11, 12]. Although there have been numerous hypotheses regarding the failure of these trials, there has been a consensus that improved brain function resulting from a medication may not be sufficient for improved learning and behavior in the absence of a parallel systematic enhancement of the learning environment. In the present study, we conducted a controlled trial of lovastatin in youth with FXS ages 10 through 17 years, combined with an open-label treatment of a parent-implemented language intervention (PILI), which has been shown to be independently efficacious when delivered to children and adolescents with FXS [13, 14]. Lovastatin is a specific inhibitor of the rate-limiting enzyme in cholesterol biosynthesis, 3-hydroxy-3-methylglutaryl coenzymeA [3HMG-CoA] reductase, and a widely used FDA-approved treatment of hyperlipidemia in children and adolescents [15]. Relevant to the treatment of FXS, lovastatin also reduces the activation of the small guanosine triphosphatase (GTPase) Ras. Consequently, activation of a signaling molecule downstream to the activation of mGluRs, specifically ERK1/2, is reduced [16]. Lovastatin has thus been considered a promising compound in the treatment of the pathophysiology of FXS.

### Pathophysiology of fragile X syndrome

The prevalence of FXS is higher in males than in females, with FXS observed in approximately 1 in every 3600 to 5000 males and in 1 in every 4000 to 6000 females [17–19]. Moreover, due to the moderating effects of the active X chromosome in females [20], males with FXS are typically more severely affected than are females with FXS. The phenotypic characteristics of FXS include hyperactivity, impulsivity, anxiety, and ASD symptomatology [21–25]. A number of researchers have argued that elucidating treatment options for the pathophysiology of FXS may provide insight into the treatment of etiologically more complex neurodevelopmental disorders, such as ASD or intellectual disability [26–28].

Remarkable advances have been made in understanding the neurobiology of FXS, and as a result, there have been dozens of investigations using pharmaceutical therapeutics to try to correct the pathophysiology of FXS. In particular, FMRP has been found to be critical for the regulation of biochemical processes involved in synaptic maturation and experience-dependent learning and is known to be expressed in mature astrocytes and in the dendrites, spines, and soma of neurons [3]. Moreover, variability in FMRP expression has been found to be associated with within-syndrome variability in cognitive performance [29].

Research findings from knockout (KO) mouse studies conducted by Bear and colleagues have led to the mGluR theory of FXS [30–32]. This theory posits that upregulation of the mGluR5 pathway contributes to multiple features of the FXS phenotype at the behavioral, electrophysiological, and molecular levels. Due to the reduction or absence of FMRP, enhanced basal protein synthesis, particularly in the hippocampus, is observed [32–34]. Numerous studies have considered the role of both mGluRs and the signaling pathways downstream of mGluR receptors as targets for treatment. Reports of treatment of the *FMR1* KO mouse with mGluR5 antagonists, as well as genetic studies in which the mGluR5-deficient mouse is crossed with the KO mouse to rescue FXS, have provided corroboration for the theory [6, 33]. Moreover, such findings suggest the possibility of rescue of the FXS phenotype at multiple levels of description, from molecular to behavioral [6, 32, 33, 35, 36].

Findings from preclinical research considering the use of lovastatin in the treatment of the phenotypic effects of FXS have been positive. In the *FMR1* KO mouse, lovastatin was observed to successfully inhibit Ras and decrease the excessive protein production [37]. The efficacy of lovastatin was assessed in a Canadian phase 1 open-label trial involving 15 individuals with FXS ages 6 to 31 years [38]. Results showed minimal side effects, indicating that lovastatin was well tolerated. Furthermore, significant improvements were observed in parent ratings of child behavior, clinical improvement ratings based on caregiver feedback, and parent reporting of adaptive functioning skills. Finally, there was a reduction in ERK activity measured in the blood that was correlated with behavioral improvements reported by the caregivers [38]. Although promising, the findings of this open-label trial must be replicated in a controlled trial to more conclusively evaluate efficacy. In the present study, we conducted a randomized controlled trial but limited our focus to individuals with FXS between the ages of 10 and 17 years, matching the age range of long-term use approved by the United States Food and Drug Administration [39]. Importantly, there is evidence from longitudinal studies of the potential for continued growth in language and cognitive skills in this age range, even for males with FXS and intellectual disability [40, 41], which suggest a need for treatment options that capitalize on that potential.

### Clinical trials in fragile X syndrome

Unfortunately, the translation of preclinical successes into human models has proved to be a challenge due to multiple limitations, such as the outcome measures used and the study design [42]. One particular challenge in the translation of preclinical findings into human trials involves the focus on behavioral changes as a key outcome. Indeed, Berry-Kravis et al. [42] hypothesized that even if a pharmaceutical agent is successful in treating underlying neural mechanisms in FXS, associated behavioral changes may be pleiotropic and/or lag neural changes. In support of this hypothesis, the use of pharmaceutical agents in the treatment of other neuropsychiatric conditions, such as ADHD, often find that using medication alone is not adequate to ameliorate these conditions [43]. In fact, findings from the Multimodal Treatment Study of Children with ADHD, a 14-month, randomized clinical trial in which behavioral and medication treatment approaches were considered alone and in combination and contrasted with community/treatment-as-usual, found that combining medication and behavioral treatment was most effective in treating functional skills such as academic achievement and social skills [40]. In a similar vein, the Child/Adolescent Anxiety Multimodal Study (CAMS [44, 45];), which is the largest randomized controlled trial for childhood anxiety disorders, found that the combination of an SSRI (sertraline) with cognitive behavioral therapy (CBT) led to better outcomes than either treatment alone [46]. In this combined study design, it is believed that the medication facilitates improved brain functioning that is most clearly demonstrated in parallel with a behavioral intervention that allows for a systematic enhancement of the learning environment [47].

In the present study, we combined our controlled trial of lovastatin with an open-label treatment of PILI, hypothesizing that if lovastatin is effective in treating the neural mechanisms underlying the FXS phenotype, the benefits of lovastatin and PILI should be greater than the benefits of PILI alone. Previous research has demonstrated the effectiveness of PILI for individuals with FXS [13, 14]. PILI aims to enhance parent use of a verbally responsive style of interaction. In this interaction style, parents [1] talk about and follow the child’s focus of attention, [2] respond to the child’s communicative overtures in affectively positive and contingent ways, [3] solicit the child’s participation in the interaction, and [4] provide examples of language that are slightly more advanced than the child’s current communicative level. There have been numerous demonstrations of the role a verbally responsive style of parental interaction can play in supporting the language development of children with FXS [48].

The presence of attentional difficulties, repetitive behaviors and/or interests, and challenging behaviors, such as frequent attempts to escape demand or social avoidance, in individuals with FXS likely makes it difficult for parents to be verbally responsive; this, in turn, can negatively impact children’s learning opportunities [49]. Parent-implemented intervention approaches have been used to successfully target parent verbal responsiveness by explicitly teaching parents how to respond to their child and navigate any challenging behaviors and characteristics [50–52]. Moreover, by teaching parents to use specific strategies for increasing their verbal responsivity, parents have the potential to use these strategies in their interactions with their children throughout the day rather than only when working with the clinicians. The benefits of parent-implemented interventions have been documented for several conditions, including autism spectrum disorder [51–53] and FXS [54, 55]. Although most of these interventions focused on young children and were delivered in the context of object play-based activities, there is evidence that older children and adolescents, including those with FXS, can also benefit from this treatment approach. In particular, McDuffie et al. [13, 14] used distance video teleconferencing (VTC) to deliver a PILI into family homes to support the language skills of males with FXS ranging in age from 10 to 17 years. Rather than deliver the intervention within the context of play, McDuffie et al. embedded the intervention within a shared storytelling context, which was hypothesized to be cognitively accessible and more age-appropriate and engaging than object play for individuals in the age range studied. These investigators also provided antecedent behavior supports to enhance language development and decrease the likelihood that challenging behaviors would occur during the shared storytelling activities.

Using both a single-case design [14] and a small-scale randomized controlled trial approach [13], it was found that mothers receiving PILI learned the targeted intervention strategies, although there was variability in the rate of acquisition and extent of the use of the responsive strategies among the treated mothers [13]. Moreover, when the parent received PILI, youth with FXS spent more time engaged in the storytelling activity and also produced more story-related utterances during the interactions [13]. Most importantly, the youth with FXS demonstrated the use of a more diverse vocabulary when the parent participated in PILI. This treatment gain in vocabulary use was also observed to generalize to a narrative language sampling context with the parent in the clinic using a storybook novel to the dyad [13]. These findings are important because [1] of the relative dearth of evidence-based nonpharmacological treatment options available for older children and adolescents with FXS, and [2] they demonstrated the continued ability of individuals with FXS to acquire new language skills well into their adolescent years.

### The present study

In the present study, a small-scale controlled trial of lovastatin was conducted in 28 youth with FXS ranging in age from 10 to 17 years. This controlled trial was combined with an open-label treatment of PILI delivered using distance VTC [13, 14]. The aims were to examine [1] the efficacy of lovastatin combined with PILI in improving child language outcomes relative to PILI alone, with a greater positive change in child language expected in the combined condition [2]; the relationship of parent use of the targeted responsive strategies to child language outcomes, with greater strategy use expected to be associated with greater change in child language; and [3] potential biomarkers of lovastatin-induced change, focusing on the cellular/molecular mechanisms by which lovastatin influences MEK/ERK and Rho GTPase signaling pathways.

## Methods

### Participants

Thirty participants (28 males and 2 females) with FXS ages 10 through 17 years were enrolled in this study. All participants had a DNA-confirmed *FMR1* full mutation. Additional inclusion criteria were [1] willingness of both the youth participant and parent/caretaker to participate in the protocol [2]; use of speech as the primary mode of communication for the youth participant, with multi-word utterances used at least occasionally [3]; youth IQ of less than or equal to 70 at screening; and [4] use of a medically acceptable method of birth control and negative serum or urine pregnancy test at screening, for sexually active female participants of childbearing potential.

Participants were excluded from the trial if any of the following criteria were met by the youth: [1] not primarily English speakers [2]; medication changes in the 4 weeks before screening [3]; major behavioral therapy or educational programming changes during the study, other than scheduled school holidays [4]; disease or condition (medical or surgical) at the screening that might compromise participant health or interfere with absorption, distribution, metabolism, or excretion of lovastatin [5]; any other reason that, in the opinion of the lead physician, rendered a youth with FXS unsuitable to participate in this study, including being unable to comply with the requirements of the study or displaying clinically significant abnormalities on safety assessments conducting during screening, use of prohibited medications per lovastatin package insert, history of recurrent status epilepticus, inability to withhold grapefruit and grapefruit juice from diet during the entire clinical trial (a factor associated with increased risk of side effects of lovastatin), unwillingness to abstain from alcoholic beverages during the trial, and active suicidality.

### Design

Primarily, participants were recruited from the Sacramento, California, metropolitan area, with an extension of recruitment nationwide to families capable of traveling to and from the clinic to complete all necessary assessments. Participants were screened initially using a pre-screening form either over the phone or in person at the clinic. Potentially eligible participants were then scheduled for a screening visit to complete assessments to confirm whether language level, current health, and overall functioning were appropriate for inclusion.

**Fig. 1:**
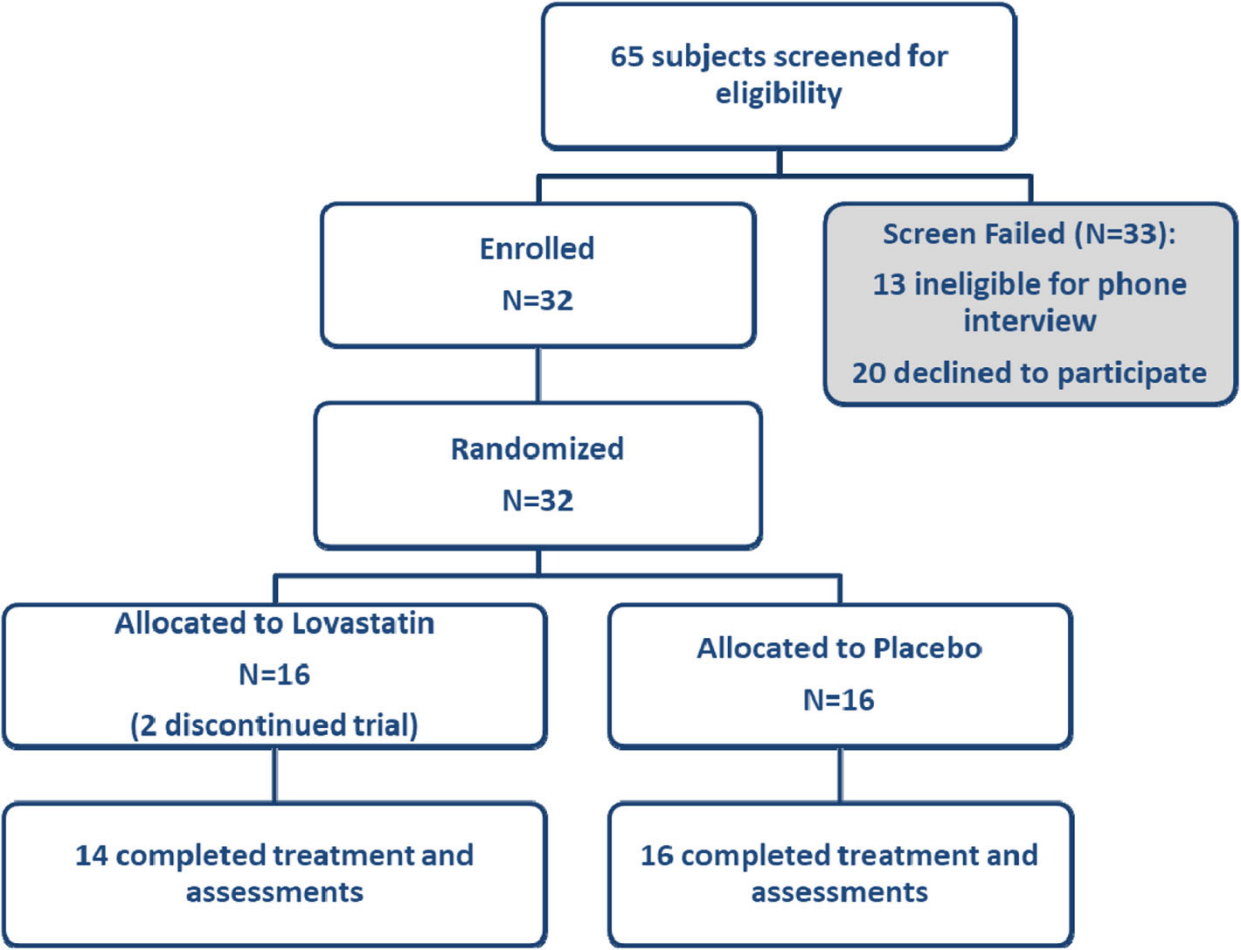
Consolidated standards of reporting trials (CONSORT) flow diagram of subject disposition

Eligible youth participants were randomized to drug or placebo in a double-blind design with all families participating in the PILI. UC Davis Investigational Drug Services carried out randomization to lovastatin or placebo via a computer algorithm (see Fig. 1). This process resulted in 14 participants assigned to the lovastatin group and 16 participants assigned to the placebo group (see Table 1). The study drug was administered orally in capsule form, starting at 10 mg of lovastatin or placebo once daily in the evening and increasing by 10 mg increments on a weekly basis or as tolerated through the first 4 weeks of the study, up to a maximum dose of 40 mg daily. Dose tolerance was assessed weekly during this titration period by phone call or email correspondence between the parent and study physician, with a single-dose decrease allowed per protocol during this period. Each participant stabilized at a maximum tolerated dose when he/she completed the 4-week titration period or after a dose decrease, whichever came first. The maximum tolerated dose was then maintained for the duration of the study. Parents were contacted approximately every other week by study staff to assess for possible adverse events and to verify proper dosing. A 10-week supply of study drug was dispensed at baseline and again at the week 10 visit. Any remaining study drug was collected at each subsequent visit to measure compliance. Compliance was also monitored using a daily dosing diary maintained by the caregiver and reviewed at each visit.

**Table 1:** Descriptive statistics of patient characteristics at pre-treatment clinic visit

Measure	Lovastatin	Placebo	***P*** value
*N*, enrolled	14	16	
*N*, incomplete	2	0	
Age	13.86 ± 2.14	13.17 ± 2.54	0.4307
Sex			0.2092
Male	12	16	
Female	2	0	
Race/ethnicity			0.05
Caucasian	14	10	
Hispanic	0	4	
African American	0	1	
Asian	0	1	
Leiter-R Brief IQ standard score	44.00 ± 9.22	43.13 ± 6.24	0.7604
Leiter-R growth score	461.40 ± 13.32	462.30 ± 8.57	0.8273
ADOS-2 comparison score	6.85 ± 2.54	6.75 ± 2.05	0.911
Parent education level			0.764
HS grad/GED	0	1	
BA/BS	5	6	
Master’s degree +	4	4	
Some college	2	5	
Some grad work	1	0	

### Parent-implemented language intervention

The PILI was delivered entirely in the participants’ homes through study-provided laptops (i.e., MacBook Air ^TM^ laptop) with pre-installed VTC (i.e., Skype) and a built-in webcam over the 20-week treatment period. These sessions were conducted by an ASHA-certified Speech-Language Pathologist (SLP) in collaboration with a Board-Certified Behavior Analyst (BCBA). A full description of the intervention can be found in McDuffie et al. [13, 14].

In the intervention, the value of verbal responsiveness and story-related talking (i.e., providing rich story-related models of vocabulary and grammar) were explained to the parent. Moreover, parents were taught how to use recasts, WH questions, and fill-in-the-blank (FIB) prompts to support their child’s active participation in the interaction (see Table 2 for the description of the strategies). The language intervention began with 2 parent education sessions delivered by an SLP that provided a rationale for the targeted strategies, as well as video examples of strategy use. The intervention then continued for approximately 12 weeks with 4 activities per week (see Table 3 for the description of activities); a break occurred during the midpoint of the treatment to allow for the week 10 onsite visit to be completed. The teaching of the parents was embedded in the context of shared storytelling using wordless picture books that the study team digitized and uploaded to an iPad Air^TM^ using the Apple iBooks^TM^ application. The picture books served as a shared conversational topic for the weekly intervention activities. The parents also received example scripts to serve as a guide when completing the shared storytelling.

**Table 2:** Description of the specific PILI verbal response strategies

Recasts	Models of a more mature version of the youth’s verbalization that provided an opportunity to learn new linguistic means of expressing the same meaning (e.g., “boy run” could be recast as “the boy is running in the yard.”). Recasts also serve to acknowledge and reinforce the youth’s conversational turn.
WH questions	Open-ended WH questions (e.g., “What is the boy doing?” and “How is the boy feeling?”) that prompt the child to provide an on-topic verbal response, engage the youth with FXS in the storytelling activity, support his/her practice of language skills, and improve the youth’s understanding of story details as well as his/her ability to retell the story in his/her own words. The youth’s responses provide an opportunity for the parent to recast the youth’s verbalization or provide story-related talking to model the appropriate response.
Fill-in-the-blank (FIB) prompts	Starting a sentence and then using a rising intonation and pause to mark an expectation that the youth with FXS is to complete the sentence (e.g., “the doggy is being chased by the …” that prompts the youth to say, “girl”). FIBs convey to the youth that an on-topic response is expected and support successful participation in the storytelling, but with minimal linguistic demands on the youth with FXS. The youth’s responses to a FIB prompt provided an opportunity for the parent to recast the youth’s verbalization or provide story-related talking to model the appropriate response.

**Table 3:** Description of the PILI sessions conducted each week

Coaching session	The clinician delivered a real-time coaching session to the parent as he/she interacted with the youth with FXS in shared storytelling. The coaching was provided to the parent via a single earbud headphone so that the child could not hear the clinician.
Homework session	The parent independently video-recorded a homework session in which he/she engaged in a shared storytelling activity with the youth with FXS using the same book as in coaching. The video was uploaded to the clinician via a secure cloud-based platform.
Feedback session	The clinician provided feedback to the parent regarding the homework in a VTC session.
Final storytelling session	The clinician observed, without coaching or intervention, a final storytelling session between parent and youth with FXS. This session was video recorded for subsequent analysis of parental strategy use and youth language and communication. A different book was used by the parent each week so that he/she gained practice in the responsive strategies and the dyad practiced the shared storytelling process rather than simply memorizing how to tell a particular story.

Prior to starting the language intervention, families completed 3 shared storytelling interactions in the home via VTC. Following these interactions, each participating parent met with a BCBA via VTC to discuss challenging behaviors that could occur during the shared storytelling. Based on this interview, each family received one behavior strategy education session during which the BCBA reviewed an individualized behavior management plan with the parent (e.g., use of reinforcement strategies, suggestions for managing challenging behaviors). This education session was followed by 3 behavior strategy practice sessions. During these practice sessions, parent-child dyads revisited the picture books used during their pre-treatment shared storytelling interactions while the BCBA provided real-time feedback via VTC regarding behavior management strategies that the parent could implement.

### Study measures

#### Safety assessments

Physical and neurological examinations, along with vital sign measurements, were completed at each visit for safety monitoring. Adverse events were documented at each visit. Standard laboratory tests of the blood and urine, including the complete blood count with differential and platelets, comprehensive metabolic panel, and lipid panel, were also performed at each visit. At baseline, molecular testing, using plasma, to confirm FXS diagnosis was carried out by PCR approach as described in Tassone et al. [56].

#### Efficacy assessment metrics

Participating caregivers completed 3 language samples with their child during both the pre- and post-treatment period. Samples were collected during a shared storytelling activity. Samples were collected over a period of approximately 8 days on average (SD = 2.73, range 4–15 days) for the lovastatin+PILI group and 7 days for the placebo+PILI group (SD = 1.51, range 2–9 days) following the pre-treatment clinic visit and over a period of approximately 4.25 days (SD = 2.45, range 2 to 9 days) for the lovastatin+PILI group and 3.9 days (SD = 3.24, range 2–14 days) for the placebo+PILI group prior to the post-treatment clinic visit. Timespan in days did not differ significantly between the 2 groups at both time points (pre: *p* = 0.2261; post: *p* = 0.7406). Three pairs of books were used for the language samples in the home (Set A: Harry and the Lady Next Door by Gene Zion, Flap Your Wings by P.D. Eastman and Strike Three, Marley! by John Grogan; Set B: Harry by the Sea by Gene Zion, The Best Nest by P.D. Eastman, and Marley’s Big Adventure by John Grogan). Book selection was counterbalanced across participants. Unlike the shared storytelling activities utilized during the intervention, the parent was not provided with scripts or clinician feedback for these samples. None of these books were used in the intervention sessions.

All samples of talk in the pre- and post-treatment storytelling interactions were recorded and transcribed by experienced transcribers using the Systematic Analysis of Language Transcripts, 2012 (SALT [57];), a software program that performs standard and customized analyses of text files that have been prepared according to a standard set of conventions that are commonly utilized in child language research (e.g., segmenting a word into root and plural morphemes: “dog/s”). The following process was used to transcribe the study samples: [1] each sample was transcribed first by a “primary” transcriber, [2] a “secondary” transcriber compared the resulting transcript against the recording and noted any perceived discrepancies, and [3] the “primary” transcriber then finalized the transcript after reviewing any discrepancies and updating the transcript as he/she deemed appropriate. Transcribers were blinded to information about the participant and when the sample was recorded (e.g., whether pre- or post-treatment).

The finalized SALT transcripts were then coded for parent story-related utterances, which included the parent’s use of the specific targeted intervention strategies (i.e., recasts, open-ended WH questions, and FIB prompts), utilizing the procedures outlined by McDuffie et al. [13]. The following outcome efficacy metrics were derived from these samples: child total number of story-related utterances, child number of different word roots (NDWR), and parent total number of story-related utterances. In addition, parental use of the specific targeted intervention strategies was considered in secondary analyses (see Table 4 for additional information on each of these metrics.) Interobserver agreement was conducted on 18 samples by computing intraclass correlations for the specific targeted intervention strategies, parent story-related utterances, and youth story-related utterances. Intraclass correlation coefficients were above 0.95 for all metrics.

**Table 4:** Efficacy assessment composite metrics computed from the three pre- and post-treatment language samples collected by the parent

Youth total number of story-related utterances	The total number of child utterances directly related to the semantic or conceptual content of the story. Repetitions and completely unintelligible utterances were not included. This metric provides an index of the total amount of child talk.
Youth number of different word roots (NDWR)	The NDWR in the first 50 story-related utterances or in the child’s total number of story-related utterances if the child produced less than 50 story-related utterances. This metric provides an index of child vocabulary size.
Parent total number of story-related utterances	The total number of story-related parent utterances. This metric includes parent’s use of the specific strategies taught in the intervention (i.e., recasts, WH-questions, and fill-in-the-blank prompts) and provides an index of the total amount of parent talk.

#### Autism Diagnostic Observation Schedule, Second Edition

The *Autism Diagnostic Observation Schedule-2* (ADOS-2) [58] is a semi-structured observational context designed to observe reciprocal interaction skills in addition to the presence of repetitive behaviors. One of four ADOS-2 modules is administered based upon the participant’s expressive language level. In the current project, this measure was completed at the pre-treatment clinic visit, and the ADOS-2 comparison score was used to describe the participant characteristics of the 2 treatment groups.

#### Nonverbal cognition

The Leiter International Performance Scales - Revised (Leiter-R [59];) is a standardized assessment of nonverbal cognition, which is administered nonverbally through the use of pantomime and gestures. The subtests comprising the Brief IQ were administered. The mean IQ in the standardization sample was 100 (SD = 15). In the current project, this measure was completed at the pre-treatment clinic visit and the Brief IQ standard score was used to describe the participant characteristics of the 2 treatment groups.

#### Language

Multiple individually administered standardized tests were included. Receptive vocabulary was assessed using the growth scores from the *Peabody Picture Vocabulary Test, 4th Edition* (PPVT-4) [60]. Expressive vocabulary was assessed using growth scores from the *Expressive Vocabulary Test, 2nd Edition* (EVT-2) [61], which was co-normed with the PPVT-4. Receptive grammar was assessed using the total number of blocks passed on the *Test for Reception of Grammar* [62]. Finally, expressive grammar was assessed using raw scores from the *Syntax Construction subtest of the Comprehensive Assessment of Spoken Language* [63]. All assessments were completed in the clinic at both the pre- and post-treatment clinic visits.

#### Behavioral rating scales

Performance on 2 behavioral rating scales was considered within analyses. First, the total score from the *Repetitive Behavioral Scale-Revised* (RBS-R [64];), an informant questionnaire designed to assess the presence of restricted and repetitive behaviors, was considered in analyses. In this measure, 43 items are rated using a 4-point Likert scale, with higher scores indicative of increased severity of repetitive behaviors. Second, the total score from the Aberrant Behavior Checklist-Community (ABC-C [65];), an informant questionnaire designed to be used to assess the presence of maladaptive behaviors, was considered. In this measure, 58 items are rated using a 4-point Likert scale ranging from 0 (not a problem) to 3 (the problem is severe in degree). These behavior rating scales were completed at the pre- and post-treatment clinic visits.

#### Clinical rating scales

Three clinical rating scales were used to assess the study clinician’s impression of youth improvements over the course of the treatment period. First, performance on the Clinical Global Impressions Scale (CGI) [66] was assessed by the study clinician at the pre-treatment clinic visit, using a Likert-scale ranging from 1 (normal) to 7 (extreme) to rate symptom severity. The level of CGI improvement was assessed by the clinician at the study midpoint as well as at the post-treatment clinic visit, using a Likert scale ranging from 1 (very much improved) to 7 (very much worse). In addition, a visual analog scale (VAS) was used to assess parental impressions of progress in two key symptoms: spoken language impairment and social impairment. Using this approach, the distance (cm) of the parent’s mark from one end of a 10-cm scale is used as the outcome variable for analysis [67–69]. Clinicians and parents were blind to whether the youth with FXS was receiving lovastatin or placebo.

#### Biomarkers’ plasma activity levels

Plasma samples were collected using EDTA-containing blood collection tubes. The blood was centrifuged for 10 min at 1000×*g* within 2 h of blood collection. The plasma was removed, aliquoted, and stored at − 20 °C. Samples were 1:20 diluted in assay buffer before testing. Plasma ERK, S6K, and MMP-9 activities were measured using the Phospho/Total ERK and Phospho/Total S6K1 2-Plex Magnetic Bead Kit and the Human MMP-Magnetic Bead Panel 2 (Merck Millipore, Billerica, MA). The preparation of plasma samples and reagents was performed according to the manufacturer’s protocol. Briefly, plates were washed with wash buffer before loading controls and samples to the appropriate wells. For MMP-9, magnetic beads, added to each well, were incubated for 2 h at room temperature followed by a washing step. Detection antibodies added to the plate were incubated for 1 h at room temperature. Streptavidin-phycoerythrin was incubated for 30 min at room temperature, and plates were washed twice with wash buffer. Sheath fluid was added to each well, and the plates were run on Luminex® (50 μL, 50 beads per bead set). For ERK and S6K1 activity levels, the plates were incubated overnight at 40 °C with shaking in the dark, following by washing, detection antibody, and incubation at room temperature for 1 h in the dark. Detection antibodies were removed and streptavidin-PE (SAPE) was added followed by a 15-min incubation at RT with shaking in the dark. The amplification buffer was added on top of the reaction mixture and the SAPE and amplification buffer were removed, and the beads were resuspended in assay buffer. Quality controls, negative and positive controls, and target samples were run in duplicates. The plates were run on Luminex® with xPONENT software. The median fluorescent intensity (MFI) data was analyzed using the spline curve-fitting method for calculating the concentrations of the markers in each sample.

#### Analysis plan

Data analyses were performed with SAS software, version 9.4 [70]. Results were expressed as mean ± standard deviation (SD) or frequencies (%). Data for each variable were examined for normality using the Shapiro-Wilk test and Kolmogorovo-Smirnov test. Not normally distributed variables were logarithmically transformed to achieve normality prior to statistical analyses. The analysis of treatment efficacy was carried out using the analysis of covariance (ANCOVA), adjusted for the corresponding baseline score as a covariate. Fisher’s exact test was applied to categorical variables. Correlations between two variables were assessed with Pearson’s correlation, and group comparisons were carried out using Fisher’s *Z* test. Student’s paired *t* tests were used to compare means before and after intervention for the same subjects within each treatment group. Adverse events were summarized by severity and relation to the drug. Two-tailed *P* values less than 0.05 were considered statistically significant as appropriate.

## Results

### Adverse events and safety assessments

Two participants (out of 30), assigned to the lovastatin+PILI group, terminated early from the study following adverse events; in both of these instances, a moderate increase in irritability was reported. No between-group differences were observed in terms of the [1] total number of participants experiencing the different types of adverse events or [2] the total number of adverse events as a function of severity (see Table 5). In addition, no relationship was established between lovastatin and the occurrence of adverse events (see Table 5). No severe adverse effects were reported during the study.

**Table 5:** Characterization of adverse events experienced by youth with FXS

Total number of participants^1^					
Treatment group	No AE	Mild event	Moderate event	Serious event	Between-group comparison
Lovastatin	1	10	1	0	0.3734
Placebo	3	9	4	0	
Total number of adverse events^2^					
Treatment Group	Mild event		Moderate event	Serious event	Between-group comparison
Lovastatin	31		1	0	0.2488
Placebo	38		7	0	
Total number of adverse events: relationship^3^					
Treatment group	Not related		Unlikely	Probably related	Between-group comparison
Lovastatin	10		12	10	0.8172
Placebo	14		21	20	

^1^Number of participants experiencing a certain severity of an adverse event where each participant is counted only once at the highest level of severity for the event

^2^Total number of adverse events experiencing a certain severity of an adverse event, including recurrence of same adverse event

^3^Total number of adverse events determined to be related to study drug (lovastatin), including recurrence of same adverse event

In addition, as would be expected when using lovastatin, a significant difference was observed in total cholesterol level between the treatment groups (*p* < .0001), with a significant decrease in total cholesterol level for the lovastatin+PILI group (baseline: *M =* 134.67, SD = 19.27; post: *M* = 104.75, SD = 17.85, *p* < .0001) over the course of the study period, but no significant change found in the placebo+PILI group (baseline: *M =* 132.27, SD *=* 19.3; post: *M* = 134.13, SD = 21.54, *p* = .652). Similarly, a significant between-group difference (*p* < 0.0001) was observed in LDL-cholesterol levels between the lovastatin+PILI group (baseline: *M =* 73.75, SD *=* 15.66; post: *M* = 47.33, SD = 14.69, *p* < .0001) and the placebo+PILI group (baseline: *M =* 70.47, SD *=* 15.35; post: *M* = 73.87, SD = 16.45, *p* = 0.368), in which participants in the lovastatin+PILI group, but not the placebo+PILI group, demonstrated a significant decrease in LDL-cholesterol levels. Finally, a between-group difference (*p* = .03) was observed in alanine aminotransferase between the lovastatin+PILI group (baseline: *M =* 22.08, SD = 10.02; post: *M* = 24.67, SD = 9.33) and the placebo+PILI group (baseline: *M =* 19.93, SD = 11.13; post: *M* = 18.13, SD = 7.75); however, significant changes were not observed during the study when groups were considered individually (lovastatin+PILI: *p* = 0.062; placebo+PILI: *p* = 0.432). In all cases, the means observed were all within the normal range.

### Treatment efficacy analyses

#### Primary outcome measures

**Fig. 2:**
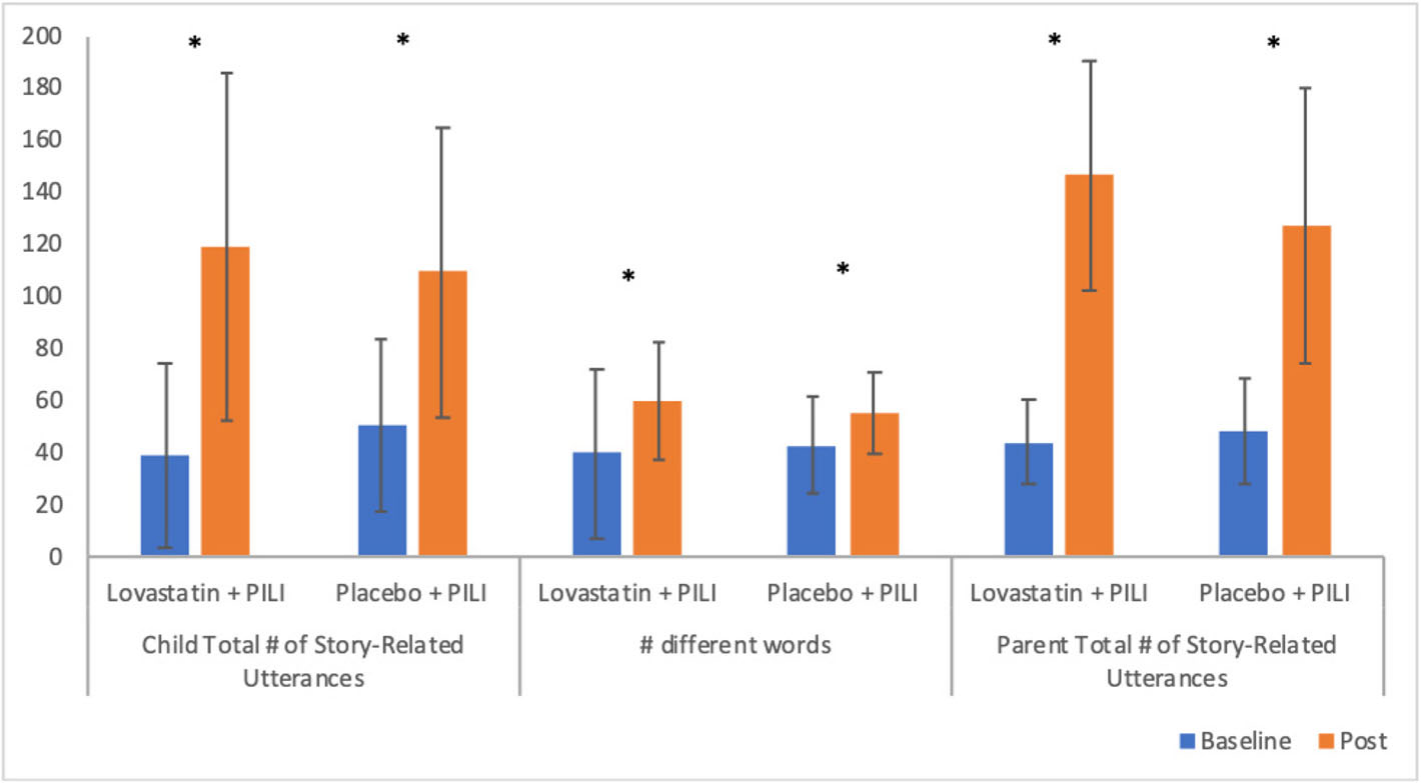
Means (with standard deviation error bars) for the primary outcome measures as a function of the treatment group. Note that all comparisons are significant (*p* < .05) after controlling for pre-treatment values

Means and standard deviations at both pre- and post-treatment for the primary outcome measures reported in this study are presented in Fig. 2 as a function of the treatment group. A significant improvement in child total number of story-related utterances, after adjusting for pre-treatment values, was observed for both the lovastatin+PILI group (*t*_(11)_ = 6.94, *p* < 0.001) and the placebo+PILI group (*t*_(11)_ = 3.92, *p* = 0.0014). No between-group difference was observed in the amount of change that occurred across the treatment period (*F*_(1, 25)_ = 0.95, *p* = 0.3379). Similarly, a significant improvement in the NDWR produced by the youth, after adjusting for pre-treatment values, was also observed both for the lovastatin+PILI group (*t*_(11)_ = 6.14, *p* < 0.001) and for the placebo+PILI group (*t*_(11)_ = 4.62, *p* = 0.0003). Again, after adjusting for pre-treatment performance, the amount of change experienced did not differ significantly between the two groups (*F*_(1, 25)_ = 2.05, *p* = 0.1644). Finally, a significant improvement in parent total number of story-related utterances used was also observed both for the lovastatin+PILI group (*t*_(11)_ = 10.31, *p* < 0.001) and for the placebo+PILI group (*t*_(11)_ = 6.94, *p* < 0.001). Again, the amount of change in parent total number of story-related utterances did not differ across the two groups (*F*_(1, 25)_ = 2.69, *p* = 0.1135) after adjusting for pre-treatment performance.

#### Secondary outcome measures

Means and standard deviations at both baseline and post-treatment for the secondary outcome measures reported in this study are presented in Table 6 as a function of the treatment group. Gains were observed in each of the 3 specific strategies taught to parents (i.e., recasts, WH questions, FIB prompts) for both the lovastatin+PILI group (recasts: *t*_(11)_ = − 8.01, *p* < 0.0001; WH questions: *t*_(11)_ = − 6.90, *p* < 0.0001; FIB prompts: *t*_(11)_ = − 5.34, *p* = 0.0002) and the placebo+PILI group (recasts: *t*_(15)_ = − 6.46, *p* < 0.0001; WH questions: *t*_(15)_ = − 4.69, *p* = 0.0003; FIB prompts: *t*_(15)_ = − 4.01, *p* = 0.0011). No between-group differences were observed in the amount of change that occurred over the course of the treatment period, after adjusting for baseline performance, in any of the strategies taught (recasts: *F*_(1, 25)_ = 2.72, *p* = 0.1117; WH questions: *F*_(1, 25)_ = 2.88, *p* = 0.1021; FIB prompts: *F*_(1, 25)_ = 0.68, *p* = 0.4158).

**Table 6:** Group comparison (mean ± SD) in secondary outcome measures between patients on lovastatin vs. placebo

Outcome	Lovastatin+PILI		Placebo+PILI	
	Baseline	Post	Baseline	Post
Receptive vocab growth score	134.75 ± 31.76	136.92 ± 33.87	137.44 ± 22.71	136.44 ± 19.79
Expressive vocab growth score	143.5 ± 22.12	142.67 ± 24.53	141.44 ± 14.45	142.75 ± 12.91
Expressive grammar raw score	10.18 ± 11.36	11 ± 12.66	8.33 ± 6.41	9.93 ± 7.59
Receptive grammar raw score	22.18 ± 18.91	22.17 ± 20.23	21.4 ± 9.26	20.2 ± 11.98
Parent strategy: recasts	3.28 ± 2.67	29.67 ± 13.34	5.48 ± 5.07	24.79 ± 12.57
Parent strategy: WH-questions	9.56 ± 9.06	40.36 ± 20.69	13.19 ± 8.57	33.25 ± 17.73
Parent strategy: fill-in-the-blank prompts	2.06 ± 2.74	19.56 ± 11.96	3.21 ± 6.96	16.5 ± 14.24
ABC-C – total score	44.5 ± 28.43	40.5 ± 31.83	55.5 ± 30.18	44.06 ± 25.36
RBS-R – total score	27.75 ± 17.43	28.67 ± 23.9	32.19 ± 18.84	26.19 ± 20.14
Clinical Global Impression (CGI)	4.58^1^ ± 0.67	2.42^2^ ± 0.67	4.50^1^ ± 0.63	2.38^2^ ± 0.72
VAS: spoken language impairment	4.06 ± 2.43	5.66 ± 1.99	1.94 ± 1.41	4.18 ± 1.53
VAS: social impairment	3.73 ± 1.74	5.18 ± 1.70	2.55 ± 1.36	3.99 ± 2.06

^1^CGI-severity ratings collected at baseline visit

^2^CGI-improvement ratings collected at post-treatment visit

Considering the direct assessments of language administered during the study period (i.e., receptive vocabulary, expressive vocabulary, receptive grammar, and expressive grammar), the performance for the lovastatin+PILI group and the placebo+PILI group was comparable over the course of the study, with neither group demonstrating significant language gains over the course of the project (*ps* ranging from 0.07–0.78).

No between-group differences were observed in the CGI-improvement ratings conducted at the post-treatment visit (*F*_1,25_) = 0.03, *p* = 0.8592), after adjusting for pre-treatment CGI-severity of these ratings. In addition, both groups demonstrated significant improvement in parent report of severity of spoken language impairment (lovastatin+PILI group: *t*_(11)_ = − 5.30, *p* = 0.003; placebo+PILI group: *t*_(11)_ = − 8.19, *p* < 0.0001) and social impairment (lovastatin+PILI group: *t*_(11)_ = − 5.80, *p* = 0.0001; placebo+PILI group: *t*_(11)_ = − 4.46, *p* = 0.0005). No between-group differences were observed in the amount of change that occurred across the groups in either of these metrics after accounting for baseline levels of performance (VAS severity of spoken language impairment: *F*_(1, 25)_ = 0.08, *p* = 0.7861; VAS severity of social impairment: *F*_(1, 25)_ = 0, *p* = 0.9781).

Significant improvements were observed in the ABC-C total scores for the placebo+PILI group, but not the lovastatin+PILI group (lovastatin+PILI group: *t*_(11)_ = 0.90, *p* = 0.3873; placebo+PILI group: *t*_(15)_ = 2.67, *p* = 0.0174); however, no between-group differences were observed in the amount of change across the treatment period (*F*_(1, 25)_ = 0.76, *p* = 0.3931). No significant improvements were observed in the RBS-R total scores for either group (lovastatin+PILI group: *t*_(11)_ = 0.26, *p* = 0.7997; placebo+PILI group: *t*_(15)_ = 1.63, *p* = 0.1231).

### Associations between parent use of treatment strategies and youth outcomes

Next, the authors considered the associations between parent total number of story-related utterances and youth outcomes. Results from these analyses indicate that percent change in parent total number of story-related utterances was significantly associated with youth gains in both total number of story utterances and NDWR produced for the placebo+PILI group, but not for the lovastatin+PILI group (see Table 7). Indeed, the strength of the association between increases in parent total number of story-related utterances and youth gains in both of these variables was significantly stronger in the placebo+PILI group than it was in the lovastatin+PILI group (*ps* < 0.046).

**Table 7:** Correlations of parent sum strategies used with a given youth outcome

Outcome	Lovastatin+PILI		Placebo+PILI		Between-group comparison
	Corr coeff. ***r***	Within-group (***H***_**0**_: ***r*** = 0)	Corr coeff. ***r***	Within-group (***H***_**0**_: ***r*** = 0)	
Child total # of utterances	− 0.095	*z* = − 0.10 (0.7742)	0.647	*z* = 0.77 (0.0054)	*z* = − 2.0 (0.0455)
Child # different words	− 0.194	*z* = − 0.20 (0.5562)	0.704	*z* = 0.88 (0.0016)	*z* = − 2.47 (0.0135)
Clinical Global Impression (CGI) - Improvement	− 0.38	*z* = − 0.41 (0.2243)	0.43	*z* = 0.46 (0.0991)	*z* = − 1.98 (0.0477)
VAS: spoken language impairment	0.306	*z* = 0.32 (0.3425)	0.299	*z* = 0.31 (0.2663)	*z* = 0.02 (0.9840)
VAS: social impairment	0.356	*z* = 0.37 (0.2643)	− 0.378	*z* = − 0.39 (0.1568)	*z* = 1.78 (0.0751)

In addition, the associations between the percent change scores between parent total number of story-related utterances and the secondary youth outcomes (i.e., CGI-I, parent report of severity of spoken language impairment, parent report of severity of social impairment) were considered (see Table 7). Of these analyses, only the associations between percent change in parent total number of story-related utterances and post-treatment CGI-I ratings were observed to differ significantly between the 2 treatment groups; however, the within-group correlations between CGI-I ratings and the percent of change in parent total number of story-related utterances observed did not meet criteria for a significant association in either treatment group.

### Biomarkers of lovastatin-induced change

Analyses were conducted to consider lovastatin-induced change in the set of biomarkers assayed, focusing on the cellular/molecular mechanisms by which lovastatin influences MEK/ERK and Rho GTPase signaling pathways. To begin, no differences were observed between the lovastatin+PILI and placebo+PILI groups in *FMR1* measures, including the percent of methylation and CGG allele category (see Table 8). Metrics of MMP9, ERK, and S6K were comparable across the lovastatin+PILI group and placebo+PILI group over the course of the study, with neither group demonstrating a significant change in any of these markers after accounting for pre-treatment measures (see Table 9).

**Table 8:** Group comparison for distributions of allele categories and % methylation for mosaic alleles between patients on lovastatin vs. placebo

Variable	Lovastatin+PILI	Placebo+PILI	Between-group comparison
**Allele category,***n* (%)			Fisher’s exact test Prob = 0.0211 (0.1717)
Full mutation	9 (75%)	7 (43.75%)	
Methylation mosaic	0	4 (25%)	
Size mosaic	3 (25%)	5 (31.25%)	
**% Methylation for mosaic alleles,** mean ± SD	85.67 ± 9.29	75.22 ± 11.98	*F*_(1, 10)_ = 1.86 (0.2026)

**Table 9:** Group comparison (mean ± SD) in molecular biomarkers between patients on lovastatin vs. placebo

Biomarker	Lovastatin+PILI			Placebo+PILI			Between-group comparison		
	Baseline	Post	Within-group: baseline vs. post	Baseline	Post	Within-group: baseline vs. post	Baseline	Post	Post, adjusted for baseline
MMP	6.36 ± 2.84	6.25 ± 2.48	*t*_(11)_ = − 0.09 (0.9313)	6.69 ± 3.65	7.20 ± 4.10	*t*_(12)_ = − 0.99 (0.3407)	*F*_(1, 24)_ = 0.01 (0.9383)	*F*_(1, 23)_ = 0.18 (0.675)	*F*_(1, 22)_ = 0.48 (0.4935)
ERK	0.48 ± 0.26	0.58 ± 0.32	*t*_(11)_ = − 0.62 (0.5502)	0.50 ± 0.22	0.84 ± 0.98	*t*_(12)_ = − 1.16 (0.2676)	*F*_(1, 23)_ = 0.16 (0.6911)	*F*_(1, 23)_ = 0.57 (0.4599)	*F*_(1, 22)_ = 0.64 (0.4332)
S6K	0.11 ± 0.10	0.11 ± 0.11	*t* (11) = 1.11 (0.2891)	0.14 ± 0.26	0.08 ± 0.04	*t*_(11)_ = 0.66 (0.5203)	*F*_(1, 23)_ = 0.08 (0.7772)	*F*_(1, 22)_ = 0.07 (0.7878)	*F*_(1, 21)_ = 0.09 (0.772)

## Discussion

Findings from preclinical research and from a phase 1 open-label trial in individuals with FXS considering the use of lovastatin in the treatment of the phenotypic effects of FXS have been positive [37, 38]. The present study was designed to provide a controlled trial of lovastatin, combined with an open-label treatment of a PILI that has been shown to be effective in youth with FXS [13]. Using this combined study design, the authors hypothesized that, if lovastatin is indeed effective in treating neural mechanisms underlying the FXS phenotype, the benefits of combining lovastatin and PILI would be greater than the benefits of PILI alone; that is, the positive effects of lovastatin would be most clearly demonstrated when combined with a behavioral intervention that allows for a systematic enhancement of the learning environment. The primary hypothesis was not supported in that no clear benefit of lovastatin over placebo was found, although additional evidence of the efficacy for PILI was obtained.

### Adverse events and safety assessments

Lovastatin is an FDA-approved statin that is widely used in the treatment of hyperlipidemia in children and adolescents [15]. This known safety profile, paired with promising findings from preclinical research considering the use of lovastatin in the treatment of the phenotypic effects of FXS, has encouraged efforts to conduct human trials to consider the efficacy of lovastatin in FXS. In the present study, lovastatin produced minimal adverse events. Moderate increases in irritability were observed for 2 participants in the lovastatin group, who then terminated early from the study. In both cases, the participants were observed to have pre-treatment irritability rating scores in the upper half of the lovastatin group score range. The total number of participants experiencing adverse events and the total number of adverse events as a function of severity did not differ significantly between the lovastatin and the placebo groups. As would be expected when using lovastatin, decreases in total cholesterol level and in levels of LDL cholesterol were observed in the lovastatin group, but not in the placebo group, with means for clinical labs in the normal range [38]. In addition, a significant difference was observed in alanine aminotransferase, an enzyme found primarily in the liver and kidney that is used to screen for/monitor liver disease, between the 2 treatment groups. Nevertheless, neither the treatment group demonstrated significant levels of change in alanine aminotransferase across the treatment period, and participant means were within the normal range. Although these data provide support for the short-term safety of statins in individuals with FXS who present with normal cholesterol levels, long-term studies are warranted. Moreover, lipid monitoring, and potentially alanine aminotransferase monitoring, should be included in any future lovastatin trial.

### Treatment efficacy

We found no significant differences in the magnitude of change observed across the study period between the participants receiving lovastatin and those receiving placebo on the primary or secondary outcome measures. Thus, we failed to replicate the findings of the open-label study conducted by Çacu et al. [38]. The different findings of the two studies may reflect the fact that our youngest participant was 10 years of age compared to 6 years in the Çacu et al. study. Indeed, it has been suggested that there is a need to test the efficacy of medication in toddlers and preschoolers with FXS to ensure maximum brain plasticity as well as to avoid the need to overcome long years of missed learning opportunities and the development of maladaptive learning strategies [42]. This explanation seems unlikely however, given that nearly half of the participants in the Çacu et al. study were over the age of 18 years. Moreover, there is evidence from longitudinal studies of the potential for continued progress in language learning during adolescence of individuals with FXS even in the absence of any systematic language intervention [40, 71]. It is also possible that the different outcome measures used in the two studies are the source of the discrepant results. The primary outcome measure for Çacu et al. was not focused on language, but on behavioral problems as assessed by the Aberrant Behavior Checklist-Community [65]. However, the ABC-C was a secondary outcome in the present study and showed no differential improvement between the placebo and lovastatin-treated participants. Thus, it may be that the gains observed in that Çacu et al. study were due to factors other than treatment with lovastatin, which cannot be ruled out given the open-label study design. In any event, the present results provide no evidence to support the use of lovastatin as a treatment for learning or behavior problems in individuals with FXS who are in late childhood or adolescence.

In contrast to the results for lovastatin, the present study provided additional evidence for the efficacy of PILI as a treatment for language problems in youth with FXS. In particular, we observed significant increases, over the course of the treatment period, in the total number of story-related utterances produced by the child, the total NDWR produced by the child, and the total number of story-related utterances produced by the parent in both the lovastatin+PILI group and the placebo+PILI group. Moreover, significant gains were observed, in both treatment groups, in each of three specific strategies taught to parents (recasts, WH questions, FIB prompts). Finally, significant improvements in parent reports of the severity of spoken language and social impairments were observed in both treatment groups. In all cases, the amount of change observed over the course of the treatment period did not differ between the two treatment groups.

Taken together, these data provide additional support that parents are able to learn the PILI-targeted intervention strategies (i.e., recasts, WH questions, and FIB prompts) with significant gains observed in the use of these strategies across a 20-week treatment period [13, 14]. In addition, as has been found in prior studies considering this PILI approach, youth participants in both treatment groups demonstrated gains in both the number of story-related utterances they produced during the shared storytelling interaction and in their diversity of vocabulary use [13]. Although the effort on the part of parents is considerable, this replication of the efficacy of PILI across multiple samples suggests that it can fill an important void for older children and adolescents with FXS, for whom there are limited evidence-based treatment options.

It is interesting to note that parent, but not clinician, ratings reflected improvements in communication—improvements that also emerged on the objective measures derived from analyses of the parent-youth shared storytelling interactions. It seems unlikely that the parent ratings were merely reflecting placebo effects given their correspondence with the objective measures. Instead, it may be that clinicians, who had only limited interactions with the youth with FXS and in a context (i.e., a clinic visit) unlikely to yield high levels of youth engagement, may simply not have had sufficient data regarding youth ability. In contrast, the parents had more broad-based observations available to them in the shared storytelling activities as well as in a variety of daily activities and thus were able to observe the real change that had occurred in their sons and daughters. These data thus suggest that parents can be accurate responders, and free of placebo effects, given access to adequate data.

Interestingly, although comparable gains in both parental use of the PILI-targeted intervention strategies and youth gains were observed in both treatment groups, parental use of the PILI strategies was correlated with youth gains in both story-related utterances and diversity of vocabulary used in the placebo group, but not in the lovastatin group. It is possible, therefore, that lovastatin may have modified or compensated for the relationship between parent use of the PILI strategies and youth gains that we were not able to directly measure in this treatment design; however, this finding needs to replicate with a new sample and is not, in and of itself, sufficient reason to prescribe lovastatin for individuals with FXS, especially because of the need for ongoing safety monitoring with lovastatin.

### Biomarkers of lovastatin-induced change

Finally, unlike some of the prior reports, MMP9, ERK1/2, and S6K1 markers were comparable across the lovastatin and placebo groups over the course of the study, with neither group demonstrating significant changes in any of the levels after accounting for pre-treatment values. The elevated MMP9 levels observed in patients with FXS enrolled in previous clinical trials were reduced in those treated with minocycline [72], which has been shown to have a high potency in MMP-9 inhibition, but they were not normalized in those treated with sertraline [73]. FMRP binds and acts as a repressor of translation of a number of mRNAs including those involved in the ERK cascade signaling pathway [74]; thus, the lack of this negative translational control is believed to lead to upregulation of these pathways and consequently to the altered synaptogenesis observed in FXS. Several studies have shown upregulation of ERK in both mouse and human (reviewed in [75]), who, contrary to our observations, recently reported a reduction of the ERK pathway signaling following a lovastatin treatment. Specifically, they reported 1.6-fold increase in basal ERK phosphorylation levels in blood platelets derived from subjects with FXS, which was rescued by lovastatin treatment and correlated with clinical response. This finding is not in line with the observations in this study as we did not detect any difference or change in phosphorylated ERK levels. The reason of this discrepancy could be due to the use of blood platelets by Pellerin and colleagues, whereas the present study utilized plasma. We opted for plasma rather than platelets because the latter requires immediate processing, which is not possible in many clinical trials, and there is a lack of consensus in the field as to how platelets should be processed in this context. Further, in addition to different sample processing methods and potential differences in expression levels among different tissues or cells, it is well known that basal ERK phosphorylation levels are very low in resting platelets giving a very low signal, which if detected and quantified with a semiquantitative approach, as is the Western blot used in the Pellerin’s study, could affect the outcome. Thus, further large studies are warranted to determine if the use of lovastatin could lead to the normalization of ERK activity and improve the FXS phenotype. Preclinical studies and in human fibroblasts have suggested that inhibitors of S6K1 should be considered for therapeutic intervention in FXS [76–79]; however, this knowledge nor this target biomarker has been successfully applied to human clinical trials.

### Limitations

There are a number of limitations to this study. As such, the present results should be considered preliminary. An important limitation is that a larger study was originally planned, with a sample size of 104 (52 per group) and projected dropout rate of 15%. That sample size would have yielded 80% power to detect a standardized effect size of 0.6 in a two-arm design for the primary outcome. Difficulty in recruitment and more limited resources in support of the project than expected led the authors to conduct the present smaller-scale study. Although speculative, our impression was that recruitment was difficult because (a) some families were resistant to using medication, especially one that is “experimental;” (b) other families were unable to commit to the study because of the time involved in terms of study visits and PILI training, especially in families in which both parents held full-time employment; and (c) there were other high-profile clinical trials recruiting simultaneously, and thus, there was a competition for families at several university sites across the country. In an attempt to remove some of the barriers to participation, we plan to examine different variants of PILI that involve added online modules, reduced synchronous coaching, and different intensities and duration of training for parents. A second limitation is that, although as discussed, there is evidence that even adolescents with FXS can continue to learn the language and so have the potential to benefit from pharmacological intervention, that potential may be greater in younger children; thus, testing of lovastatin in a younger cohort would be useful in the future before abandoning it as a treatment option. A third limitation of the study is that the small number of females and non-Caucasian participants in the study limits generalizability. Finally, a longer study design is needed to better understand if any long-term problems are associated with the use of lovastatin in youth with FXS who present with normal cholesterol levels. At the same time, long-term post-treatment follow-up is needed to determine whether the benefits of PILI are maintained after the intervention of the clinicians has ceased. In fact, one of the arguments in favor of changing parent behavior is that the parents can, hopefully, maintain the new behavioral style and so their sons and daughters will continue to benefit after the clinician-delivered training has ceased. However, we have yet to document maintenance of PILI benefits either for parent or child. Long-term follow-up of PILI is now in progress.

## Conclusions

The present study evaluated a 20-week controlled trial of lovastatin in youth with FXS, paired with an open-label treatment of PILI, to assess whether the benefits of lovastatin combined with PILI would be greater than the benefits of PILI alone. Significant improvements were observed in all primary outcome measures, and in parent report of severity of spoken language and social impairments, in both treatment groups. Across the treatment period, comparable amounts of change were observed in both treatment groups. Although the hypothesis that the benefits of lovastatin and PILI would be greater than the benefits of PILI alone was not supported, data from the present project provide additional support for the efficacy of the use of PILI in youth with FXS.

## Data Availability

The datasets used and/or analyzed for the present paper and can be made available upon a reasonable request to the corresponding author.

## Abbreviations

ABC-C: Aberrant Behavior Checklist-Community
ADOS-2: Autism Diagnostic Observation Schedule-2
ANCOVA: Analysis of covariance
ASD: Autism spectrum disorder
BCBA: Board-Certified Behavior Analyst
CGI: Clinical Global Impression
ERK1/2: Extracellular signal kinase
EVT-2: Expressive Vocabulary Test, 2nd Edition
FIB: Fill-in-the-blank
FMRP: Fragile X mental retardation protein
FXS: Fragile X syndrome
GTPase: Guanosine triphosphatase
KO: Knockout
Leiter-R: Leiter International Performance Scales – Revised
MFI: Median fluorescent intensity
mGluRs: Metabotropic glutamate receptors
NDWR: Number of different word roots
PILI: Parent-implemented language intervention
PPVT-4: Peabody Picture Vocabulary Test, 4th Edition
RBS-R: Repetitive Behavioral Scale-Revised
SALT: Systematic Analysis of Language Transcripts
SAPE: Streptavidin-PE
SD: Standard deviation
SLP: Speech-Language Pathologist
VAS: Visual analog scale
VTC: Video teleconferencing
